# The Left Atrial Appendage in Sinus Rhythm and Atrial Fibrillation: From Functional Structure to Potential Thromboembolic Reservoir, Rationale for Medical or Radical Exclusion

**DOI:** 10.3390/jcm15010284

**Published:** 2025-12-30

**Authors:** Jacob Zeitani, Ermal Likaj, Marco Stefano Nazzaro, Alban Dibra, Kolja Sievert, Horst Sievert

**Affiliations:** 1 Neurosciences and Rehabilitation Department, University of Ferrara, 44121 Ferrara, Italy; 2 Cardiac Surgery Department, Mother Teresa University, 020983 Tirana, Albania; 3 Cardiology Department, S. Camillo Hospital, 00100 Rome, Italy; 4 Cardiology Department, Mother Teresa University, 020983 Tirana, Albania; 5 Cardiovascular Center Frankfurt, 60389 Frankfurt, Germany

**Keywords:** atrial fibrillation (AF), device occlusion, direct oral anticoagulation (DOAC), left atrial appendage (LAAO), surgical excision, thrombus, transcatheter

## Abstract

The left atrial appendage (LAA) is a highly dynamic anatomical structure that plays a key role in left atrial reservoir function, pressure and volume modulation, and endocrine hormone secretion during sinus rhythm. However, its physiological contribution is profoundly altered in atrial fibrillation (AF). Electrical and structural remodeling, impaired contractility, and blood stasis within the LAA collectively transform this functional component into the principal cardiac source of thrombus formation and embolic events in patients with AF. This review focuses on the conceptual continuum from physiological LAA function in sinus rhythm to its pathological transformation in AF and the evolving rationale for progressively more complete (“radical”) anatomical exclusion A variety of strategies, including systemic anticoagulation therapy, percutaneous device-based exclusion, and surgical closure, are currently employed, each with specific indications, limitations, and procedure-related risks. Beyond summarizing available techniques, this review critically synthesizes mechanistic, anatomical, and clinical data to address unresolved controversies regarding patient selection, residual leaks, device-related thrombosis, and post-procedural antithrombotic management. Finally, emerging directions toward minimizing residual foreign material, reducing thrombogenicity, and achieving durable exclusion are discussed, supporting a more personalized and radical approach to stroke prevention in AF.

## 1. Introduction

The left atrial appendage (LAA) plays an essential role in maintaining normal cardiac physiology during sinus rhythm, contributing to left atrial reservoir function, volume regulation, and endocrine signaling [1,2,3]. However, clinical attention toward this structure intensifies markedly with the development of atrial fibrillation (AF). In AF, profound alterations in electrical activation, mechanical contractility, and intracavitary blood flow transform the LAA from a functional, highly dynamic atrial component into the predominant cardiac source of thromboembolism [4,5]. AF is associated with an approximately five-fold increase in ischemic stroke risk, and more than 90% of left atrial thrombi in non-valvular AF originate from the LAA. Although this association was firmly established in clinical trials during the 1970s and 1980s, effective stroke-prevention strategies were initially limited, leaving patients exposed to substantial thromboembolic risk [6,7]. Clot formation reflects a complex interplay between LAA anatomy, atrial mechanical dysfunction, and the patient’s clinical risk profile, as captured by contemporary risk stratification schemes such as the CHA_2_DS_2_-VA score, in which female sex is no longer considered an independent determinant of thromboembolic risk [8,9,10]. Traditionally, systemic oral anticoagulation has represented the cornerstone of stroke prevention in AF. However, bleeding risk, contraindications, intolerance, and poor long-term adherence leave a substantial proportion of patients insufficiently protected [11]. This therapeutic gap has driven the development of mechanical strategies aimed at eliminating the LAA as a thromboembolic reservoir. Percutaneous and surgical LAA exclusion were initially conceived as alternatives for patients unable to tolerate anticoagulation but have progressively evolved toward broader clinical application, including opportunistic surgical closure during cardiac surgery and increasingly sophisticated transcatheter approaches [12,13,14]. Despite expanding clinical use, several fundamental questions remain unresolved: how much physiological LAA function can be sacrificed in pursuit of stroke prevention; which anatomical, functional, and clinical features should guide the choice between anticoagulation, percutaneous occlusion, or surgical exclusion; and how clinicians should interpret randomized trial data in relation to real-world registries that include older, frailer, and more complex patients. Accordingly, the primary aim of this review is not merely to summarize devices or clinical trials, but to integrate embryological, anatomical, functional, and clinical insights into a translational conceptual framework linking mechanism to patient selection and therapeutic strategy, with particular emphasis on emerging directions toward more complete and less thrombogenic (“radical”) LAA exclusion.

## 2. Embryology and Physiology of the Adult Left Atrial Appendage

The LAA, before becoming a potential thromboembolic reservoir in the AF patient, represents the embryologic remnant of the primitive left atrium and develops early in cardiac morphogenesis. During the fourth week of gestation, the common atrial chamber gives rise to two lateral outpouchings that form the right and left atrial appendages. As the pulmonary veins and their tributaries are progressively incorporated into the posterior wall of the left atrium, the original embryonic cavity becomes confined to the appendage. Consequently, the LAA retains the characteristic trabeculated structure formed by pectinate muscles, in contrast to the smooth-walled body of the definitive left atrium derived from pulmonary venous tissue [1].

After birth, although no longer essential for chamber formation, the LAA preserves several physiological roles. It contributes to left atrial compliance and functions as a dynamic reservoir that modulates left ventricular filling, particularly under conditions of volume or pressure overload. Moreover, the LAA has important endocrine properties; it contains the highest concentration of atrial natriuretic peptide (ANP) and brain natriuretic peptide (BNP) granules within the atria [2,15,16]. Stretch-induced secretion of these peptides promotes natriuresis, vasodilation, and inhibition of the renin–angiotensin–aldosterone system, thereby playing a critical role in cardiovascular homeostasis.

However, aging and atrial fibrillation are associated with progressive structural remodeling, fibrosis, and reduced appendage compliance, accompanied by a decline in ANP content, granule density, and stretch-induced secretory responsiveness [3,13]. BNP regulation in this context increasingly reflects pathological wall stress rather than preserved endocrine function [14]. As a result, the LAA’s reservoir and conduit functions decline, reducing its contribution to left ventricular filling and atrial pressure buffering [17]. Impaired appendage contractility further promotes blood stasis, providing a mechanistic explanation for the increased thromboembolic risk observed in older patients and those with AF.

While surgical or percutaneous exclusion of the appendage is an established strategy for stroke prevention, the intentional elimination of a structure with residual physiological, endocrine, and compliance-related functions continues to raise important questions regarding the long-term consequences of increasingly complete (“radical”) LAA exclusion, particularly in younger patients or those with lower baseline thromboembolic risk.

## 3. From Physiologic Function to Potential Thrombus Formation

Loss of atrial contractility, reduced appendage emptying velocities, blood flow stasis, LAA complex architecture, and cardiovascular risk factors collectively render the LAA the predominant site of thrombus formation, accounting for over 90% of left atrial thrombi in non-valvular AF [5]. As a result, AF increases stroke risk five-fold, with a worse prognosis than strokes of other etiologies.

### 3.1. Left Atrial Appendage: Morphology and Clinical Relevance

Variability in LAA morphology significantly influences hemodynamics, flow stasis, and risk of thrombus formation [6]. Di Biase et al. classified LAA into four main types, each associated with different levels of thromboembolic risk:

- Chicken Wing (lowest risk): Characterized by a sharp bend or fold in the dominant lobe, often with a “hooked” appearance. This morphology is associated with reduced stasis and lower thromboembolic risk.
- Windsock (intermediate risk): A single dominant lobe of variable length with minimal branching. Blood flow dynamics are moderately favorable, conferring intermediate risk.
- Cactus (higher risk): Multiple lobes extending from a central body, resembling a cactus. The branching architecture promotes regions of slow flow and higher thrombus risk.
- Cauliflower (highest risk): A short, complex structure without a clear dominant lobe. Its irregular anatomy and low flow velocity predispose to marked blood stasis, higher prevalence of spontaneous echo contrast (“smoke”), and the greatest risk of thromboembolic events [7].

Interestingly, thrombi are more frequently formed in the LAA than in the right atrial appendage (RAA) among patients with AF, a phenomenon thought to reflect differences in luminal surface area, appendage volume, and neck geometry between the two structures. Both RAA and LAA are trabeculated with pectinate muscles largely running parallel to each other with a comb-like appearance, although these characteristics are less pronounced in LAA [15,16]. Yamaji et al. found that all atrial thrombi were attached to pectinate muscles [17]. Their study showed that the RAA has fewer pectinate muscles, a smoother surface, and a smaller volume than the LAA, which may explain its lower susceptibility to thrombus formation. It has also been shown that in patients with AF, the endocardium of the LAA exhibits an elevated expression profile of prothrombotic and proinflammatory proteins compared with the right atrial appendage, indicating increased thrombogenicity of the LAA compared with RAA [18]. This may explain, at least in part, the observation that most clots are formed in the LAA in patients with AF. These morphological and biological characteristics have important implications for procedural planning, device selection, and the risk of incomplete exclusion or peri-device leak during LAA occlusion.

### 3.2. CHA_2_DS_2_-VASc: Stroke Risk Assessment in Atrial Fibrillation

As the management of atrial fibrillation has evolved, one lesson has remained constant: not all patients with AF share the same risk of stroke. For decades, clinicians struggled with how best to identify those who would truly benefit from anticoagulation therapy or LAA occlusion. Early attempts, such as the CHADS_2_ score, offered a helpful starting point but left far too many patients in a gray zone, individuals who were labeled “intermediate risk” yet still suffered preventable strokes [19]. From this clinical uncertainty emerged the need for a more sensitive, more granular approach, leading to the development of the CHA_2_DS_2_-VASc score, which has become the most widely adopted tool for thromboembolic risk stratification [8,9].

The strength of the CHA_2_DS_2_-VASc score lies in its recognition that stroke risk is multifactorial and extends beyond a few dominant comorbidities. Instead of considering age as a threshold, the score acknowledges the progressive nature of risk by assigning additional weight to those aged 75 or older while still capturing the substantial, but previously overlooked risk in individuals aged 65–74. It also includes vascular disease and the subtle but important influence of female sex, which acts not as an isolated risk factor but as a modifier when other comorbidities are present [20].

Heart failure and reduced ventricular function promote blood stasis; hypertension and diabetes accelerate endothelial dysfunction; vascular disease embodies the burden of atherosclerosis; and a prior stroke or TIA, the most heavily weighted element, serves as a potent reminder that cerebrovascular disease rarely gives only one warning.

One of the most important contributions of the CHA_2_DS_2_-VASc score is to definitively identify patients at low risk [9]. This is crucial because anticoagulation, while highly effective, is not without bleeding hazards. With older scoring systems, too many patients were placed into an ambiguous “intermediate” category, where uncertainty often translated into undertreatment or overtreatment. In clinical practice, the CHA_2_DS_2_-VASc score is interpreted across three major risk categories that guide decisions regarding anticoagulation. Patients at truly low risk are those with a score of 0 in men or 1 in women, where the only point may be attributed to sex; in this group, the annual stroke rate is so low that anticoagulation offers no meaningful benefit. The intermediate-risk category includes men with a score of 1 and women with a score of 2, a zone where the decision to initiate therapy requires individualized consideration, integrating bleeding risk, comorbidities, imaging markers, AF burden, and patient preference. Beyond this level, the risk of thromboembolism rises steeply: men with scores of 2 or more and women with scores of 3 or more fall into the high-risk category, for whom long-term oral anticoagulation is clearly recommended unless contraindicated. As the score increases, the patient/year risk of thromboembolism rises consistently, providing a clear and evidence-based rationale for recommending anticoagulation, especially with today’s safer direct oral anticoagulants.

Despite its broad clinical utility, the CHA_2_DS_2_-VASc score has recognized limitations. It does not incorporate anatomical variables such as LAA size, morphology, or flow characteristics, nor does it account for AF burden or duration, factors increasingly linked to thromboembolic risk. Reflecting these considerations, recent European Society of Cardiology (ESC) guidelines recommend the use of the non-sex-based CHA_2_DS_2_-VA score for routine stroke risk assessment (Level of Evidence C), acknowledging that female sex functions primarily as an age-dependent risk modifier rather than an independent risk factor [11].

In summary, while the LAA contributes little to adult cardiac physiology, it plays an outsized role in the thromboembolic complications of AF. Clinical risk stratification using CHA_2_DS_2_-VA identifies patients who benefit from systemic anticoagulation, but it does not fully capture the anatomical and functional determinants of LAA thrombogenicity. Consequently, the choice between pharmacological anticoagulation and mechanical LAA exclusion has become a central and increasingly individualized question in contemporary AF management.

## 4. Anticoagulation Therapy

To prevent stroke in patients with atrial fibrillation, risk stratification based on the CHA_2_DS_2_-VA score identifies those who derive a clear net benefit from long-term oral anticoagulation. Accordingly, most patients with AF and elevated thromboembolic risk are recommended to receive lifelong anticoagulant therapy. Historical data demonstrated that adjusted-dose warfarin reduced stroke risk by approximately 60%, whereas antiplatelet therapy achieved only modest risk reduction (~20%) and is no longer recommended for stroke prevention in AF [21].

Vitamin K antagonists, once the mainstay for preventing thromboembolism in AF, have largely been replaced by DOACs, which offer a better risk–benefit profile and eliminate the need for routine monitoring. Large randomized clinical trials have demonstrated that DOACs are non-inferior to warfarin for ischemic stroke prevention and are associated with a significantly lower risk of fatal and intracranial hemorrhage, establishing them as the preferred oral anticoagulants for the majority of eligible AF patients [22,23,24,25].

Regardless of the type of oral anticoagulant used, potential drug, food, and supplement interactions should be taken into consideration and include this information in patient education, as both VKAs and DOACs have notable interaction risks [26,27]. Although DOACs have fewer interactions than VKAs, they are not entirely interaction-free. CYP3A4, involved in factor Xa inhibitor metabolism, interacts with many drugs, while P-glycoprotein modulates DOAC transport and is affected by several cardioactive agents such as digoxin, calcium channel blockers, and antiarrhythmics [28,29,30]. The ESC provides guidance on managing these combinations, though real-world data remain limited [11]. Genetic polymorphisms, particularly in the CES1 gene, may increase dabigatran levels and bleeding risk, while variations affecting factor Xa inhibitors appear to have minimal clinical impact [31,32]. Despite major advances in systemic anticoagulation, bleeding risk remains a central limitation of long-term therapy and represents a frequent cause of treatment discontinuation or underuse. To estimate bleeding risk, the HAS-BLED score has been proposed as a clinical risk stratification tool, used to estimate the 1-year risk of major bleeding in patients with AF who are being considered for oral anticoagulation [33]. Developed from the Euro Heart Survey cohort, the score incorporates common clinical variables and provides a standardized framework for assessing bleeding risk in routine practice. The score includes the following parameters, each contributing one point to the total score: Hypertension (systolic blood pressure > 160 mmHg); Abnormal renal and/or liver function (defined as chronic dialysis, renal transplantation, serum creatinine ≥ 200 µmol/L, or chronic liver disease with significant biochemical abnormalities); Stroke (previous ischemic or hemorrhagic stroke); Bleeding history or predisposition (including previous major bleeding or intracranial hemorrhage); Labile INR (time in therapeutic range < 60% in patients treated with vitamin K antagonists); Elderly (age ≥ 65 years); and Drugs or alcohol (concomitant use of antiplatelet agents or NSAIDs, or excessive alcohol consumption). A HAS-BLED score ≥ 3 identifies patients at high risk of bleeding; however, this classification does not constitute a contraindication to anticoagulation. Instead, the score highlights the need for closer clinical monitoring and correction of modifiable risk factors [34]. Elements such as uncontrolled hypertension, concomitant use of interacting medications, poor INR control, and excessive alcohol intake represent actionable targets for risk reduction. When used alongside stroke-risk assessment tools such as CHA_2_DS_2_-VASc, the HAS-BLED score supports individualized decision-making and promotes safe optimization of anticoagulant therapy in patients with AF [35,36].

Finally, several studies have demonstrated that patients’ adherence to long-term oral anticoagulation is suboptimal, with a marked decline after the first year of therapy. This reduced adherence is influenced by multiple factors, including concerns about bleeding, medication fatigue, lifestyle limitations, and the asymptomatic nature of atrial fibrillation itself [37,38]. As adherence wanes, the protective effect of anticoagulation diminishes, leaving patients exposed to an increased risk of thromboembolic events. In this context, non-pharmacological strategies for stroke prevention, particularly mechanical exclusion of the left atrial appendage, have emerged as an important alternative for selected patients, offering durable protection without reliance on lifelong medication adherence [39].

## 5. Surgical and Percutaneous Left Atrial Appendage Exclusion

Patients who are unable to tolerate long-term anticoagulation because of major bleeding events, high bleeding risk, or absolute contraindications represent a particularly challenging population in AF management. For these individuals, the benefit of systemic anticoagulation is often outweighed by the risk of recurrent or life-threatening bleeding, leaving them insufficiently protected from thromboembolic events [40,41].

In this context, left atrial appendage (LAA) exclusion, surgical or percutaneous, has emerged as an important alternative strategy for stroke prevention. Both approaches aim to mechanically eliminate the primary site of thrombus formation, thereby reducing stroke risk without requiring continuous anticoagulant therapy.

### 5.1. Surgical Left Atrial Appendage Closure

The concept of surgically removing or occluding the left atrial appendage (LAA) originated in 1946, when William Dock proposed its resection as a preventive measure against recurrent systemic emboli in patients with rheumatic mitral stenosis and atrial fibrillation. Soon after, Hellerstein demonstrated in dogs that atrial appendectomy was technically feasible and followed by complete endothelialization without thrombus formation. The first human LAA resections, reported by Madden in 1949, were performed as prophylaxis against recurrent embolic events, marking the first clinical application of LAA exclusion [42]. These early procedures coincided with the emergence of open-heart surgery in the late 1940s and early 1950s, representing the beginning of a new era in cardiac surgery where direct intracardiac interventions became feasible and reproducible.

Approximately one-third of patients undergoing cardiac surgery have a documented history of AF, which is associated with an increased risk of stroke and mortality [43].

**Figure 1:**
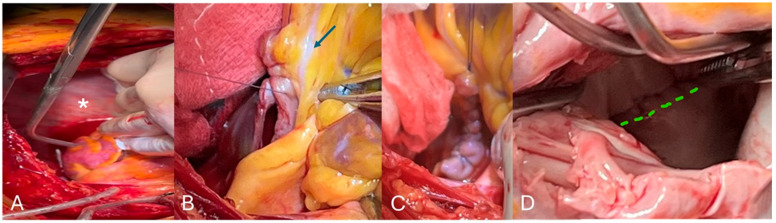
Intraoperative view of the left appendage * (**A**) amputated and initial surgical closure, the circumflex artery is indicated with an arrow (**B**), completed surgical closure (**C**), and the occluded appendage orifice from the left atrium view is shown with a dotted line (**D**).

Different surgical strategies have been proposed, including ligation, resection, or LAA orifice suturing when the left atrium is opened (Figure 1). Among these, surgical excision provides the most consistent and durable closure, whereas ligation or stapler-based methods are more susceptible to incomplete sealing, especially in anatomically complex appendages [44,45,46]. Multiple lobes or intricate morphologies, particularly in the cranial region near the pulmonary veins, may impede complete exclusion and allow residual flow, thereby diminishing stroke prevention efficacy [46,47]. The LAAOS III trial demonstrated that surgical exclusion during cardiac surgery reduces embolic events in patients with atrial fibrillation, independent of anticoagulation status [48].

Stand-alone epicardial LAA closure represents an alternative for AF patients unsuitable for either anticoagulation therapy or percutaneous LAAO. Observational studies have demonstrated its feasibility, procedural safety, and potential role in this high-risk subset [49]. A preoperative 3D model based on CT-Scan might help with surgical planning and device selection.

### 5.2. Transcatheter Left Atrial Appendage Occlusion

**Figure 2:**
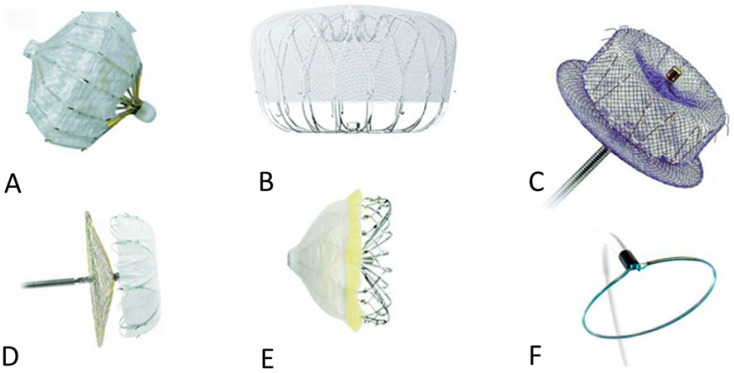
Left appendage closure devices: (**A**)—PLAATO, (**B**)—Watchman, (**C**)—Amulet, (**D**)—Lamber, (**E**)—Wave Crest, and the epicardial device (**F**)—LARIANT. Large clinical trials, including PROTECT-AF and PREVAIL, established non-inferiority to warfarin for stroke prevention in nonvalvular AF, supporting its use in patients with contraindications or intolerance to long-term anticoagulation [55,56,57,58]. Based on clinical experience and new technologies, new generations have been introduced with the latest Watchman Flex.

Left atrial appendage occlusion has emerged as an important therapeutic option for stroke prevention in patients with non-valvular AF who are unsuitable for long-term oral anticoagulation (OAC) [43,50]. The concept of percutaneous LAA occlusion emerged in the late 1990s, adapting principles from surgical ligation for catheter-based delivery. H. Sievert was among the early pioneers of LAAO, contributing substantially to its development in the late 1990s and early 2000s [12]. Sievert played a key role in the first clinical evaluations of the PLATOO device (Appriva Medical, Sunnyvale, CA, USA), helping to establish foundational principles of device design, implantation technique, and procedural safety (Figure 2A) [51]. These early efforts laid important groundwork for the subsequent evolution of modern LAAO technologies. The first extensively studied device demonstrated procedural feasibility with high short-term closure rates. Although later withdrawn, PLAATO provided a proof of concept that informed subsequent device development. Building on this foundation, the WATCHMAN device (Atritech, later Boston Scientific, Plymouth, MN, USA) became the first transcatheter LAA occluder to receive FDA approval in March 2015 (Figure 2B). Its self-expanding nitinol frame with a permeable polyester membrane seals the LAA orifice while permitting endothelialization [52,53,54].

Another major advancement in percutaneous LAA occlusion is represented by the Amplatzer Cardiac Plug (ACP) and Amulet devices (Abbott, formerly AGA Medical, Plymouth, MN, USA) [59]. These devices use a lobe-and-disk design to accommodate variable LAA anatomies, enhancing sealing in complex appendages (Figure 2C). The Amulet device, in particular, offers greater flexibility in sizing and positioning, extending applicability to patients whose anatomy may not be suitable for the WATCHMAN system [60,61,62]. Other nitinol-based devices, including the LAmbre LAA closure system (Lifetech Scientific, CA, USA), have shown high procedural success (Figure 2D), minimal periprocedural complications, and nearly complete LAA sealing at follow-up. The WaveCrest LAAO system (Figure 2E) (Biosense Webster, CA, USA) and Occlutech occluders have demonstrated favorable procedural safety and success, receiving CE Mark approval in 2013 and 2016, respectively [63,64].

Additional devices, such as Sideris Patch, Ultraseal, SeaLA, LeFort, and Pfm, remain under preclinical or early clinical investigation [65]. Epicardial approaches, exemplified by the Lariat system (Figure 2F), utilize combined endocardial and epicardial access with magnetic wire guidance to deploy a lasso suture around the LAA, achieving both mechanical closure and electrophysiological isolation [66]. Collectively, these developments highlight the transition of LAA occlusion from traditional surgical ligation to minimally invasive, anatomically adaptable transcatheter and epicardial approaches. However, in catheter-based LAAO, the presence of an LAA thrombus constitutes a procedural contraindication. Anticoagulation for at least four weeks is recommended to achieve thrombus resolution before intervention, and the procedure should only proceed once thrombus clearance is documented.

### 5.3. Anticoagulation After Percutaneous and Surgical Left Atrial Appendage Closure

According to the guidelines, LAAO is the alternative strategy for stroke prevention in patients with atrial fibrillation who are at high risk of bleeding or have contraindications to long-term anticoagulation. Therefore, bleeding complications remain a concern, particularly during the intensive antithrombotic phase aimed at preventing device-related thrombosis (DRT) [67]. Indeed, post-procedural anticoagulation remains challenging, as many patients are at elevated bleeding risk or cannot tolerate anticoagulation. Current recommendations advise oral anticoagulation (VKA or DOAC) plus aspirin for 45 days, followed by dual antiplatelet therapy (DAPT) with aspirin (81–325 mg) and clopidogrel (75 mg) for six months, and aspirin alone indefinitely thereafter [68].

For patients with absolute contraindications to OAC, DAPT for six months, as demonstrated in the ASAP study, or a shorter course of 1–3 months in high bleeding-risk individuals may be considered. This strategy also allows optimal management of concomitant coronary artery disease using DAPT or low-dose DOAC with single antiplatelet therapy [69].

Although randomized evidence is limited, observational studies and expert consensus (EHRA/EAPCI) support the safety and efficacy of LAAO in patients who cannot receive temporary OAC [70]. Major bleeding occurs in approximately 5–10% of patients within the first year after LAAC and is an independent predictor of mortality. In select high-bleeding-risk patients who cannot tolerate DAPT or short-term OAC, single antiplatelet therapy (SAPT), or, in rare cases, no antithrombotic therapy, may be considered. Across registries, 5–10% of patients received SAPT, or no therapy post-LAAC, and DRT incidence was low in the EWOLUTION and Amulet studies [71]. However, these studies are limited by small sample sizes and selection bias. In the RELEXAO registry, patients discharged without antithrombotic therapy experienced higher DRT rates compared with treated patients (15.4% vs. 4.5%; *p* = 0.02) [72]. Therefore, SAPT or no therapy should be reserved for carefully selected patients with prohibitive bleeding risk [34,35].

Unlike percutaneous LAA occlusion, the optimal strategy for anticoagulation management and remnant imaging after surgical LAA exclusion is not well established. Trials evaluating the beneficial effect of surgical LAA closure in patients undergoing cardiac surgery but without a known history of AF are ongoing [72].

Pitfalls and Long-Term Considerations.

### 5.4. Device-Related Complications

Although contemporary devices and procedural experience have markedly improved safety and success rates, transcatheter LAAO remains subject to important technical and clinical pitfalls that can influence both early procedural outcomes and long-term efficacy.

A key challenge is appropriate patient selection; suboptimal selection may expose patients to procedural risk without providing a greater net clinical benefit. Equally important is pre-procedural imaging, typically involving transesophageal echocardiography (TEE) and increasingly cardiac CT, which provides high-resolution assessment of LAA morphology, landing zone dimensions, lobar complexity, and thrombus exclusion [73,74]. Inadequate imaging can lead to incorrect device choice or sizing, failed deployment, or even procedural cancellation.

Transseptal puncture further contributes to variability in outcomes; improper puncture location, particularly excessively superior or anterior entry, may restrict catheter maneuverability and impede coaxial alignment with the LAA. Similarly, inaccuracies in landing-zone measurements may result in device undersizing, predisposing to instability and peri-device leak, or oversizing, increasing the risk of wall trauma or incomplete expansion.

Among post-implant complications, peri-device leak (PDL) represents one of the most clinically significant pitfalls. PDL occurs when the device fails to achieve complete circumferential sealing of the LAA ostium, an issue more common in anatomically complex appendages such as cauliflower-type, multilobulated, or shallow LAAs [75,76]. While small leaks (<3 mm) are often considered benign, moderate or large leaks have been associated with reduced stroke-prevention efficacy and a higher risk of device-related thrombus (DRT). Preventive strategies involve meticulous multimodality imaging, appropriate transseptal puncture, accurate device sizing, and adherence to PASS criteria (Position, Anchor, Size, and Seal), before release [77].

Device migration, although rare, represents a potentially life-threatening complication. Migration typically results from inadequate anchoring due to under sizing, insufficient device compression, or incomplete engagement of fixation mechanisms [78]. Acute embolization may present with hemodynamic compromise if the device obstructs the mitral valve or embolizes into the aorta, whereas subacute migration can lead to distal embolization into peripheral arteries. Management may require percutaneous retrieval or surgical intervention, depending on device location and patient stability. Strict adherence to sizing algorithms, imaging-guided alignment, and stability testing during deployment are critical to minimizing migration risk [79].

Beyond the early procedural period, post-procedural antithrombotic management remains a major determinant of long-term outcomes. Inadequate adherence to recommended antithrombotic regimens, whether OAC, dual antiplatelet therapy, or modified protocols for high-bleeding-risk patients, can contribute to DRT, especially in the presence of residual leaks or incomplete endothelialization. Indeed, despite continuous refinement of LAAO design and implantation techniques, DRT remains a clinically relevant concern. Although relatively uncommon, with incidence typically being reported as being between 1.6–16% depending on patient profile and imaging surveillance, the presence of DRT is strongly associated with a significantly increased risk of ischemic stroke and systemic embolism [70,71,72,73,74,75,76,77,78,79,80,81,82,83]. Its development is classically explained through Virchow’s triad, in which blood stasis, surface thrombogenicity, and patient-specific hypercoagulability converge at the device–blood interface. In patients with persistent AF, reduced atrial and appendage mechanical function promotes static flow conditions around the device. Incomplete endothelialization, particularly over metallic structures, and procedural factors such as deep implantation or residual peri-device leaks may further exacerbate local shear abnormalities and predispose to thrombus formation. Beyond procedural and device-related contributors, patient characteristics exert a substantial influence. High thromboembolic risk scores, impaired ventricular function, renal dysfunction, advanced age, and underlying prothrombotic conditions have all been linked to a greater propensity for DRT [84]. Anatomical features, including large, multilobed, or trabeculated LAA morphology, may create niches where slow flow persists even following device deployment. Importantly, premature interruption or intolerance of post-procedural antithrombotic therapy remains one of the key modifiable predictors of early thrombus formation. Most DRT events are identified within the first three to six months following implantation, coinciding with the period of tissue healing and endothelial coverage. For this reason, structured imaging follow-up using TEE or cardiac CT during this phase is essential. When detected, prompt intensification of anticoagulation is typically effective and recommended until complete resolution is documented. Preventive strategies focus on optimizing every step of the pathway: meticulous patient selection, attention to device sizing and positioning, and personalized antithrombotic regimens that balance bleeding vulnerability against thrombosis risk. Advances in surface technologies, such as reduced metal exposure and bioactive or polymeric coatings, are expected to lessen thrombogenicity and shorten the duration of pharmacologic protection required after implantation. As such innovations continue, the ultimate goal remains to achieve durable LAA exclusion while minimizing the risk of thromboembolic complications and enhancing overall procedural safety. Finally, scheduled follow-up imaging, typically at 45–90 days, is essential to identify PDL progression, late device migration, or DRT formation. Failure to perform structured imaging has been linked to missed diagnoses and preventable embolic events [85,86].

Collectively, these pitfalls emphasize that optimal outcomes in LAAO rely on careful patient selection, high-quality imaging, precise technical execution, and standardized follow-up pathways. By addressing these vulnerabilities, operators can maximize procedural success and maintain the long-term efficacy of LAAO in contemporary clinical practice.

## 6. Discussion

Atrial fibrillation continues to rise in prevalence worldwide, largely driven by population aging and the growing burden of cardiovascular comorbidities. This epidemiologic trajectory underscores the urgent need for effective, individualized stroke-prevention strategies as in AF patients more than 90% of cardiac thrombi in non-valvular AF originate in the LAA and associated with a five-fold increase in ischemic stroke risk [5]. The LAA is a highly variable and complex cardiac structure, whose size and shape have significant functional and clinical implications [1,6]. Its dimensions, length, width, and orifice size, reflecting differences in both hemodynamic behavior and thromboembolic risk. The LAA size is influenced by atrial pressure and rhythm, typically enlarging in patients with atrial fibrillation and chronic left atrial remodeling. Imaging studies, echocardiography, cardiac CT, and MRI show that larger LAA dimensions particularly increased ostial diameter and depth are associated with reduced contractile function and slower emptying velocities, both recognized as markers of blood stasis and thrombogenic potential [7]. Furthermore, the shape of the LAA classified as “chicken wing,” “windsock,” “cactus,” or “cauliflower” is closely linked to its size, with more complex or multilobed morphologies often correlating with greater volume and higher risk of thrombus formation. Understanding these characteristics is essential for risk stratification and procedural planning. Thus, in both sinus rhythm and atrial fibrillation, “size and shape truly matter”.

The interplay between appendage anatomy, impaired contractile function, and intraluminal stasis creates a markedly thrombogenic environment. While CHA_2_DS_2_-VA stratifies systemic stroke risk, it does not fully capture LAA-specific anatomic or functional determinants of thrombogenesis. Traditional stroke-prevention strategies in atrial fibrillation rely on clinical risk scores and pharmacological therapy, missing the mechanistic link between atrial remodeling, LAA dysfunction, and thromboembolism. Structural atrial disease, characterized by left atrial enlargement, fibrosis, impaired contractility, and altered flow dynamics, creates conditions of blood stasis and endothelial dysfunction that promote thrombogenesis, often before sustained AF becomes clinically evident. Thus, the LAA evolves from a functional reservoir to a thromboembolic nidus. Patient selection for preventive strategies should therefore integrate multiple domains beyond rhythm status and age-weighted scores. These include (i) the extent of atrial remodeling and dilation, (ii) LAA morphology and flow characteristics, (iii) the anticipated lifetime risk of AF recurrence or progression, and (iv) bleeding vulnerability as reflected by tools such as the HAS-BLED score.

Mechanical exclusion of the LAA represents an anatomical solution to an anatomical problem, with distinct implications depending on the chosen modality. Percutaneous LAA occlusion provides an alternative for patients unsuitable for anticoagulation, though it carries risks such as device-related thrombus, peri-device leaks, and post-procedural antithrombotic requirements. Surgical LAA exclusion performed concomitantly with open-heart procedures allows direct visualization, durable closure, and elimination of foreign material from the atrial cavity, thereby maximizing long-term embolic protection when complete exclusion is achieved.

In younger patients undergoing mitral valve repair, these considerations gain particular relevance. For patients with significant atrial remodeling and complex LAA morphology, prophylactic surgical LAA exclusion may provide durable stroke prevention while minimizing cumulative bleeding risk. For selected patients with significant atrial remodeling, complex LAA morphology, and anticipated long-term exposure to anticoagulation, prophylactic surgical LAA exclusion may offer a rational preventive strategy that balances durable stroke prevention against cumulative bleeding risk. This mechanistic, patient-centered framework shifts decision-making from reactive treatment toward anticipatory cerebrovascular protection.

Oral anticoagulation remains the cornerstone of stroke prevention in AF, with both vitamin K antagonists and DOACs demonstrating substantial risk reduction [11]. Yet, contraindications, intolerance, and adherence issues leave a subset of patients insufficiently protected, creating a therapeutic gap. In such cases, the risk of thromboembolism persists despite the inability to use systemic anticoagulation, creating a therapeutic gap in contemporary practice [87,88,89].

Left atrial appendage exclusion, either percutaneous or surgical, provides a direct mechanical strategy aimed at eliminating the nidus for thrombus formation. Clinical trials and real-world registries have established LAA occlusion as an effective alternative for patients who cannot tolerate long-term anticoagulant therapy [45,50]. Devices such as Watchman, Amulet, and WaveCrest demonstrate favorable outcomes, though device-related thrombosis, peri-device leaks, and post-procedural antithrombotic needs remain challenges [65,67,69]. These limitations highlight an important area of unmet need: the development of next-generation LAA devices that minimize foreign material, reduce inflammatory and thrombogenic surfaces, and simplify post-procedural pharmacotherapy. Strategies that incorporate atraumatic anchoring, conformable structures, eventually bioresorbable components, or reduced-profile implants could meaningfully lower the risks of DRT and leakage while expanding eligibility to patients who cannot safely receive any form of anticoagulation after implantation. Radical exclusion, complete anatomical separation with minimal residual material, represents a promising future direction. Given the expanding prevalence of AF in an aging population, combined with the persistent risk of thromboembolism and the limitations of both pharmacologic and current mechanical strategies, innovation in LAA exclusion is both timely and necessary. Prophylactic surgical LAA exclusion performed opportunistically during open-heart procedures is also gaining attention. Opportunistic LAA excision may reduce lifetime thromboembolic risk and reliance on systemic anticoagulation, particularly in patients likely to undergo future interventions Eliminating the LAA at the time of open surgery provides durable protection against thromboembolism and reduces long-term dependence on systemic anticoagulation. Among available surgical approaches, complete excision of the LAA with direct suturing of the atrial wall offers the most definitive and durable closure, minimizing the chance of residual stump or incomplete exclusion [14]. This anatomical eradication of the appendage provides greater certainty than clips, stapling, or external ligation. However, excision and suturing may increase the risk of perioperative bleeding, particularly in fragile atrial tissue or in the context of complex concomitant procedures. Despite this, many surgeons consider excision the gold standard when feasible, as it achieves the highest completeness of closure, an essential advantage for patients likely to undergo future structural heart interventions.

Taken together, the evidence reviewed supports a shift from a purely score-driven, reactive approach to stroke prevention toward a mechanism-based and personalized strategy centered on the LAA. A practical framework therefore integrates clinical risk, LAA morphology, atrial remodeling, rhythm burden, bleeding susceptibility, and feasibility of durable exclusion. Table 1 summarizes LAA morphology, thromboembolic risks, and suggested therapy.

From a practical standpoint, oral anticoagulation remains the first-line therapy for most patients with AF; however, mechanical LAA exclusion should be actively considered in patients with contraindications, intolerance, poor adherence, or anticipated long-term bleeding risk. In patients undergoing cardiac surgery, opportunistic surgical LAA exclusion, preferably by complete excision, offers a definitive, one-time intervention that may reduce lifetime thromboembolic risk and future dependence on anticoagulation. In non-surgical candidates, percutaneous LAA occlusion provides an effective alternative, though careful attention to anatomy, device selection, and post-procedural antithrombotic management is essential.

The overarching take-home message is that the LAA is not merely a passive anatomical structure but a modifiable therapeutic target. Early, complete, and durable exclusion, when appropriately selected, represents a rational evolution toward radical, anatomy-based stroke prevention, particularly in an aging population with increasing AF prevalence and cumulative bleeding risk. Aligning patient-specific mechanisms with tailored therapeutic strategies may ultimately redefine long-term cerebrovascular protection in AF.

## 7. Conclusions

Oral anticoagulation remains central to stroke prevention in atrial fibrillation; yet contraindications, intolerance, or poor adherence leave some patients unprotected. For these individuals, mechanical exclusion of the LAA provides a rational alternative by directly addressing the dominant anatomical source of thromboembolism. This review supports a paradigm shift from a purely score-driven strategy toward an anatomy and mechanism-based approach to stroke prevention. By combining systemic risk assessment, atrial remodeling, LAA anatomy, and bleeding risk, clinicians can tailor therapy for maximal, durable protection. Future advances aimed at achieving complete exclusion with minimal thrombogenic burden may further refine cerebrovascular protection and optimize long-term management of atrial fibrillation in an aging population.

## Figures and Tables

**Table 1:** LAA Morphology, Thromboembolic Risk, and Therapy.

LAA Morphology	Thromboembolic Risk	Flow/Function	Preferred Intervention	Post-Procedural Antithrombotic Strategy
Chicken Wing	Low	Good emptying	OAC or Watchman	Standard OAC/DAPT
Windsock	Intermediate	Moderate flow	OAC or Watchman/Amulet	OAC 4–6 wks → DAPT 3–6 mo
Cactus	High	Slower emptying	Amulet/Epicardial LAAO	DAPT 3–6 mo or tailored SAPT
Cauliflower	Very High	Severe stasis	Amulet/Epicardial LAAO/Surgical excision	Individualized; high bleeding risk → SAPT

## Data Availability

No new data were created or analyzed in this study. Data sharing is not applicable to this article.
